# The Role of Mast Cells in the Induction and Maintenance of Inflammation in Selected Skin Diseases

**DOI:** 10.3390/ijms24087021

**Published:** 2023-04-10

**Authors:** Ewelina Woźniak, Agnieszka Owczarczyk-Saczonek, Magdalena Lange, Justyna Czarny, Ewa Wygonowska, Waldemar Placek, Bogusław Nedoszytko

**Affiliations:** 1 Department of Dermatology, Sexually Transmitted Diseases and Clinical Immunology, The University of Warmia and Mazury, 10-229 Olsztyn, Poland; 2 Department of Dermatology, Venereology and Allergology, Medical University of Gdansk, 80-214 Gdansk, Poland; 3 Invicta Fertility and Reproductive Centre, Molecular Laboratory, 81-740 Sopot, Poland

**Keywords:** mast cell activation, urticaria, Kounis syndrome, Ehlers–Danlos syndrome, pseudoallergy, red man syndrome, psoriasis, rosacea, atopic dermatitis, allergic contact dermatitis, cutaneous mastocytosis, mast cell activation syndrome

## Abstract

Under physiological conditions, skin mast cells play an important role as guardians that quickly react to stimuli that disturb homeostasis. These cells efficiently support, fight infection, and heal the injured tissue. The substances secreted by mast cells allow for communication inside the body, including the immune, nervous, and blood systems. Pathologically non-cancerous mast cells participate in allergic processes but also may promote the development of autoinflammatory or neoplastic disease. In this article, we review the current literature regarding the role of mast cells in autoinflammatory, allergic, neoplastic skin disease, as well as the importance of these cells in systemic diseases with a pronounced course with skin symptoms.

## 1. Introduction

Mast cells (MCs) are mononuclear cells originating from pluripotential hematopoietic cells in the bone marrow. MCs are derived from the myeloid lineage as granulocytes, monocytes, erythrocytes, and megakaryocytes [1]. Progenitors with mast-cell-forming potential are defined as CD34+, KIT+, FcεRI + cells [2]. According to some authors, the integrin α4β7 mediates the migration of MC progenitors to peripheral tissues, where they finally differentiate into mature cells [3]. The mastocyte progenitor cell transforms into a mature MC ultimately in the peripheral tissue under the influence of various factors of a specific microenvironment. The diversification, growth, and maturing of MCs in tissues may take between several days and up to even several weeks [4].

MCs can be divided into two groups, depending on the secreted granularity: MCs(T)—MCs releasing only tryptase—and MCs(TC)—MCs secreting, in addition to tryptase, chymase, caboxypeptidase, and a cathepsin G-like proteinase. MCs(T) are founded in the mucous membranes of the respiratory system and the digestive tract, that is, in places inside of the body where MCs come into contact with factors from the outside world (e.g., food, pollen, drugs, microorganisms). However, MCs(TC) localize in the submucosa and connective tissue adjacent to the conjunctiva and skin [5,6].

Due to the strategic location of MCs in tissues adjacent to the external environment, they physiologically play a role in the processes of innate and acquired immunity against microbes, as well as stimulate tissue healing after trauma. Moreover, MCs are involved in the pathogenesis of many allergic, autoinflammatory, and cancer diseases [7].

MCs express a lot of different membrane receptors and molecules, and are capable of producing a wide range of mediators, cytokines, and chemokines, with pro- or anti-inflammatory effects.

Tryptase is a serine protease stored in basophil granules and MCs, secreted outside the cell, trigger the inflammatory cascade. Experiments in animals confirmed the role of tryptase in promoting inflammatory cell recruitment, vascular permeability, and airway hypersensitivity and remodeling [8]. Studies show that an elevated basal tryptase level (BTL), defined clinically as > 11.4 ng/mL, affects 4–6% of the general human population [9,10]. The increased BTL may be associated with mastocytosis, renal failure, and hereditary alpha tryptasemia, and may also appear as a certain individual feature without a diagnosed disease [11].

In order to better understand the importance of MCs in various skin diseases, we decided to take a closer look at the pathophysiology of autoinflammatory, allergic, neoplastic diseases and systemic syndromes with skin manifestations.

## 2. Mast Cells and Their Role in the Skin

The skin is the largest organ in the human body and plays a protective role against external factors such as physical, chemical, and microbiological. The presence of various cells of the immune system in the skin makes it able to effectively protect itself against microorganisms and react with healing to injury. One of the representatives of the immune system are MCs, which are involved in both innate and adaptive immune response [12].

The majority of MCs in the skin are MCs(TC) which occur in the greatest density in the superficial dermal zone and are mostly located near blood vessels and nerves, where they can react quickly [13].

One of the more well-known mechanisms of MC activation is the IgE-mediated reaction, the type I hypersensitivity reaction, which is associated with a rich symptomatology. The allergen–IgE–receptor FcεRI connection leads to the release of enzymes contained in MCs within a few minutes, which results in vascular dilatation and increased vascular permeability, which in the skin directly causes swelling, erythema, pruritus, and the formation of urticarial wheals. In the case of extensive reactions, mediators released from MCs dilate large vessels, leading to anaphylactic shock, which can be fatal [14]. This type of reaction more often occurs in patients with atopic dermatitis and urticaria.

In addition, MCs can be activated by IgG immunoglobulins through FcγRI- and FcγRIIa. MC stimulation by interferon gamma (INFγ) causes the formation of IgG receptors on the surface of the cell membrane, and accumulation of this receptors leads to cell degranulation and the generation of metabolites of arachidonic acid and the secretion of chemokines and cytokines [15]. Among the external factors activating the non-IgE-dependent pathway of MCs, there are also drugs or microbial antigens, e.g., lipopolysaccharide (LPS) or bacterial toxins [16].

Due to the fact that MCs in the skin are located close to sensory nerves, they can be endogenously stimulated by neuropeptides such as neurotensin (NT), nerve growth factor (NGF), substance P (SP), pituitary adenylate cyclase-activating polypeptide (PACAP), and vasoactive intestinal polypeptide (VIP). MCs themselves can also synthesize SP or NGF, suggesting an autocrine or paracrine mechanism [17,18,19]. High concentrations of SP cause the degranulation of MCs. SP in combination with an interleukin-1 (IL-1) family member, interleukin-33 (IL-33), increases the secretion of pro-inflammatory cytokines such as tumor necrosis factor α (TNF- α) and IL-1β [20]. VIP mediates the activation of MCs through vasoactive intestinal peptide receptor 2 (VPAC2) and/or Mas-related G-protein couplet receptor member X2 (MRGPRX2) receptors. Together, SP and VIP release cytokines and chemokines by MCs, including TNF-α, granulocyte–macrophage colony-stimulating factor (GM-CSF), interleukin-3 (IL-3), C-C motif chemokine ligand 2 (CCL2), C-C motif chemokine ligand 5 (CCL5), C-X-C motif chemokine ligand 8 (CXCL8/IL-8), C-X-C motif chemokine ligand 9 (CXCL9), and C-X-C chemokine ligand 10 (CXCL10) [21].

MCs can also be activated through psychological stress. This happens through the hormones of the hypothalamus–pituitary–adrenal axis. On the surface of MCs there is mainly corticotropin-releasing hormone receptor 1 (CRH-R1). Corticotropin-releasing hormone (CRH) causes vasodilation in the skin, but the mechanism of action is not clear. On the one hand, CRH has a vasodilatory effect but also activates MCs, whose granules also have the same effect on blood vessels [22]. NT stimulates human MCs to release vascular endothelial growth factor (VEGF) and augments the effect of CRH on VEGF specific to NT, but this mechanism is not yet clear [23].

MCs are also the source of renin and constitute a unique non-renal renin–angiotensin system. Moreover, they contain angiotensin II and manifest gene expression of the renin–angiotensin system [24,25].

**Figure 1:**
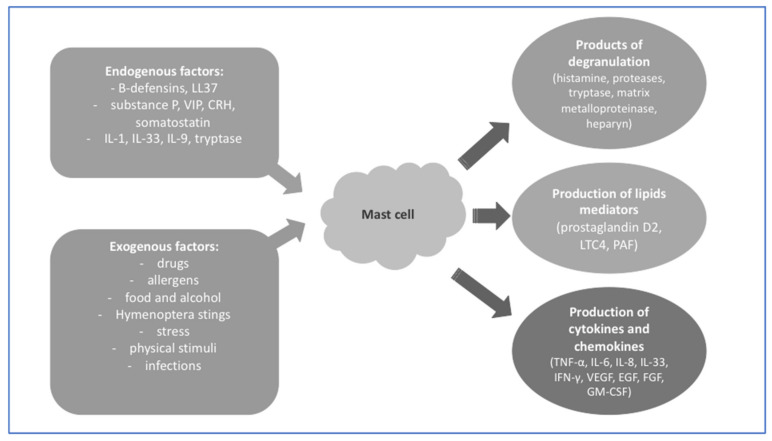
Activity of MCs [26,27,28].

Figure 1 presents the internal and external factors which may activate MCs for degranulation and the release of enzymes, as well as the production of inflammation mediators [26,27,28].

## 3. Mast Cell Activation Syndrome—Why It Is an Important Disease for Dermatologists?

The term “mast cell activation syndrome (MCAS)” refers to a group of disorders of different pathogenesis, manifested through episodic multisystemic symptoms as a result of MC mediators being released. MCASs are defined by systemic severe and recurrent mast cell activation (MCA), usually in the form of anaphylaxis, a substantial, event-related increase of the serum tryptase level beyond the individual’s baseline and a response of the symptomatology to drugs directed against MCs, mast-cell-derived mediators, or mediator effects [29].

Genetic factors, environmental factors, and susceptibility to atopy are factors predisposed to the development of MCASs (Table 1) [29]. The coexistence of many factors in one person increases the risk of MCASs, for example, severe types of MCASs occur more often when the patient simultaneously suffers from hereditary alpha tryptasemia (HαT), systemic mastocytosis (SM), and an IgE-dependent allergy (often against Hymenoptera venom) [29].

Signs of MC activation may be local (flush, erythema, pruritus, urticaria, local angioedema, rhinitis, sneezing, cough, dyspnea, diarrhea) or systemic—mild (mild hypotension without shock, mild headache, cough, mild dyspnea, mild diarrhea, loose stool, nausea, and mild abdominal cramping), severe (anaphylactic shock, sweats, fever, unconsciousness, severe skin problems, hypoxia, wheezing, exacerbation of asthma, acute cramping, vomiting, and diarrhea), and chronic (tissue inflammation/atopic tissue inflammation, chronic skin problems, diarrhea and/or nausea, cough, fatigue, depression, and other neuropsychiatric symptoms) [29].

The diagnosis of mast cell activation syndrome (MCAS) is established when three of the following criteria are present simultaneously:

1. typical clinical symptoms arising from recurrent acute systemic MC activation (resembling recurrent anaphylaxis) have been documented;
2. MC-derived mediators increase substantially in the serum (tryptase) or urine (histamine or prostaglandin-D2 metabolites) over the individual’s baseline (standard test: documented increase in serum tryptase levels following the 120% + 2 ng/mL formula);
3. The symptoms respond to drugs blocking the activation of MCs, MC mediators, mediator production, or mediator effects [29,40,56,57].

Depending on the etiology, MCAS can be divided into three major types:

- primary (clonal = monoclonal)—characterized by the detection of *KIT* D816V mutation, MCs aberrantly display CD25 in most cases with confirmed mastocytosis (CM or SM), with only two minor SM criteria;
- secondary (reactive)—characterized by an IgE-mediated allergy, another hypersensitivity reaction, or another immunologic disease that can induce the activation of MCs;
- idiopathic—the criteria to diagnose MCAS are met, but no related reactive disease, no IgE-dependent allergy, no hereditary alpha tryptasemia, and no neoplastic MC are found [29].

Researchers in recent years have been proving that two or three variants of this syndrome (mixed form of MCAS) may coexist in one patient, which increases the risk of severe anaphylaxis [29,58,59]. Treatment strategies are personalized for each patient, depending on the symptoms and their etiology [29].

## 4. Role of Mast Cells in Autoimmune Diseases

MCs influence the development of autoimmune diseases in humans, which is confirmed by numerous animal models. MCs may take part in the pathomechanisms of these diseases at several stages:

- they stimulate T lymphocytes through the expression of MHC class II, ensuring direct co-stimulation through the expression of receptors on the surface of the cell;
- they influence the increased proliferation and activation of T lymphocytes through various produced cytokines;
- they influence the maturing, migration, and functions of dendric cells and may have an indirect impact on the function of T cells;
- they reverse the Treg suppression of T effector cells and reduce the susceptibility of T effector cells to the Treg suppression;
- they recruit neutrophiles [60].

Infection or immunosuppression are believed to be the reasons behind the development of MCAS in autoimmune diseases, and the pathomechanism itself of the disease has not been discovered fully [61,62]. The role of MCs in the pathogenesis of sclerosis multiplex, rheumatoid arthritis, bullous pemphigoid, and diabetes type I has been proven [60].

## 5. Role of Mast Cells in Psoriasis

Psoriasis is a group of autoinflammatory diseases, in which we distinguish psoriasis vulgaris with or without psoriasis arthritis and pustular psoriasis. Those diseases have different pathophysiology and skin manifestations. In this section, we will focus on the role of MCs in the pathophysiology of psoriasis vulgaris (PV).

PV is a chronic skin disease, which is characterized by the appearance of well-defined erythematous plaques on the skin covered with silvery white scales. Psoriatic plaques are typically located on the straight surfaces of the elbows and knees, the scalp, and the lumbosacral region. However, the disease can occur anywhere in the skin, including the matrix of the nails and nail bed. Nail matrix psoriasis is indicated by pitting, leukonychia, red spots in the lunula, and nail plate crumbling. Nail bed psoriasis is characterized by onycholysis, splinter hemorrhages, oil drops, discoloration, and hyperkeratosis.

PV can appear at any age, with two peaks at ages 18 to 39 years and 50 to 69 years [63]. Genetic factors play a role in the development of this disease. Studies on the genome of patients with PV have shown an increased risk of this disease in the presence of alleles (*HLA-Cw6*, HLADQ* 02:01, *CCHCR1*, and *CYP1A1*) and loci (*PSORS1-9* and *PSORSAS*) [64,65]. The use of algorithm-termed minimum-distance-based enrichment analysis for genetic association (MEAGA) showed 87 significantly enriched functions/pathways; many of these are immune-related functions such as lymphocyte differentiation/regulation, Type I interferon, pattern recognition, response to stimulus, response to virus/bacteria, regulation of the I-κB kinase/NF-κB cascade, and regulation of the adaptive immune response [64]. The course of psoriasis is characterized by periods of remission and exacerbation. Several environmental and behavioral factors are known to provoke or exacerbate skin lesions, e.g., skin trauma, infections, stress, smoking, and certain medications such as lithium and interferon [66,67].

The pathogenesis of psoriasis is still under investigation. Current data prove the central role of the adaptive immune system. The cells mainly involved in the formation of inflammation are T-helper cell type 1 (Th1), T-helper cell type 17 (Th17), plasmacytoid dendritic cells, natural killer T cells, macrophages, and keratinocytes [67]. The role of MCs in the pathogenesis of PV is still being discussed.

### 5.1. Inflammatory Response to Injury—The Koebner Phenomenon

The Koebner phenomenon in psoriasis is the appearance of lesions after a mechanical injury, e.g., after a scratch, tattooing skin, radiations, skin incision, viral infections, striae, etc. [68]. Already in the 1980s, researchers Toruniowa and Jablonska showed the presence of MCs in the skin of patients with psoriasis, determining their number from 30 min to 14 days after the irritation of healthy skin. The significant difference was noticed from the 4th day onwards after scratch, reaching a peak at day 14, simultaneously with the appearance of Koebner’s phenomenon, which was observed in 74% of patients with active disease and in 37.5% with stationary psoriasis. Moreover, in contrast to normal wound healing, the number of MCs steadily increases at the time of formation of the earliest psoriatic lesions [69]. The mechanism of the formation of psoriatic lesions in the Koebner phenomenon takes into account the role of mast-cell-derived inflammatory mediators such as tryptase, IL-6, IL-8, IL-17, and IL-36γ, and the increased expression of NGF and vascular endothelial growth factor (VEGF). Moreover, there is the key role of the α 2 β1 integrins, S100A7 (psoriasin) and S100A15 (koebnerisin), change in the ratio of CD4^+^/CD8^+^ T cells, downregulation of mechanosensitive polycystin 1 protein, decrease in inflammation-controlling atypical chemokine receptor 2 (ACKR2), and the reduced expression of N-methyl-d-aspartate (NMDA) receptors (NMDARs) on the keratinocytes, and increased levels of chemokines (CXCL8 and CCL20) in inducing the formation of new psoriatic lesions [68].

### 5.2. Response to Stress—Neuroinflammation in Psoriasis

MCs are localized close to blood vessels and peripheral nerves in the skin, where they probably play an important role in the response to stimuli: environmental (e.g., mechanical trauma, changes of temperature) and psychological (e.g., different ways to deal with emotional stress) [70]. In recent years, the answer to the question of how stress affects the development of psoriasis has been sought. Researchers are looking for links between the brain, the peripheral nervous system, the endocrine system, and the immune system [71].

The epidermis is innervated by a three-dimensional network of unmyelinated fine nerve fibers with free branching endings that arise in the dermis. Epidermal nerves have been shown to contain SP, neurokinin A (NKA), and calcitonin gene-related peptide (CGRP), whereas dermal nerves secrete SP, CGRP, VIP, and NKA [72,73]. The source of neuropeptides in the skin can also be keratinocytes or cells of the immune system. In the skin, neuropeptides can induce MCs to release vasoactive amines that facilitate the infiltration of neutrophils and T cells, and can modulate the immune system during psoriasis development [74].

Interestingly, in patients suffering from psoriasis, it was observed that accidental damage to the skin nerves after injuries of the cutaneous sensory nerves contributed to the remission of psoriatic lesions [75,76].

## 6. Role of Mast Cells in Atopic Dermatitis

The activation of MCs constitutes a key element in the development of symptoms of atopic dermatitis (AD). AD is a genetically determined, chronic, recurring skin inflammatory disease with complex pathogenesis. The characteristic for most patients is increased IgE in the serum, which correlates with the severity of the disease. IgE contributes to the development of AD through intermediation in the activation of MCs located in the skin, induced by various allergens. Similarly, as in MCAS, the basic immunological mechanism of MCA in this disease is the immune response consisting of the activation of these cells through binding specific IgE with receptor FcεRI on their surface. Activated MCs excrete three classes of substances:

- preformed chemical and protein mediators, such as histamine, serotonin, heparin and chondroitin sulphates, proteases, acid hydrolases, cathepsin, etc.;
- lipid mediators, such as prostaglandins, leukotrienes, and the platelet-activating factor (PAF);
- preformed and/or newly synthesized growth factors, cytokines, and chemokines, such as TNF-α, transforming growth factor beta (TGF- β), macrophage inflammatory protein-1a (MIP-1a/CCL3), monocyte chemoattractant protein-1 (MCP-1/CCL2), VEGF, IFN-α/β/γ, GM—CSF, IL-1a/b, IL-2, IL-3, IL-4, IL-5, IL-6, IL-8, IL- 9, IL-10, IL-11, IL-12, IL-13, IL-15, IL-16, IL-18, and IL-25 [77].

Through the cytokines, chemokines, and growth factors produced, MCs may regulate the recruitment, transport, and functions of cells taking part in the skin immune response in AD. For example, through the tumor necrosis factor alfa (TNF-α), IL-4 and IL-13 induce cell adhesion molecules on the endothelium which may participate in the recruitment of leukocytes. MCs may also modulate the differentiation of naïve T lymphocytes to Th1 and Th2, enhancing activation of lymphocytes [78]. They may also regulate the development of primary B cells and stimulate the synthesis of IgE in B cells [77,78]. Finally, MCs may directly present antigens to T lymphocytes. Thus, they may function both as effector cells and regulatory cells; as a result of which, they are of key relevance in the pathogenesis of AD [78]. Research by Kanbe, T. et al. showed that the degranulation of MCs is correlated with the stage of advancement of AD. The comparison of the levels of the soluble stem cell factor of MCs, SCF, and its KIT receptor in the serum of 54 patients with AD, 5 patients with ordinary psoriasis, and 64 healthy people showed that levels of SCF and the KIT receptor are correlated with the exacerbation of the disease in AD patients but not in patients with psoriasis [79]. The role of the activation of MCs in AD has been confirmed by tests of MC products in the skin and plasma of patients with this disease. The skin and plasma of AD patients contain higher concentrations of histamine, as well as Il-4 and Il-13. It has been shown that MCs are one of the main types of cells manifesting the expression of these interleukins in AD [78]. The role of MCs in AD has been also confirmed by tests on transgenic mice which, when exposed to the action of Il-4 or Il-13, developed skin changes similar to AD in the epidermis [80].

## 7. Role of Mast Cells in Kounis Syndrome in the Progression of Urticaria

In the progression of acute urticaria or exacerbated chronic urticaria, Kounis syndrome may develop. It is a complex, multisystem, and multiorgan clinical reaction of the arterial system (particularly of coronary, mesenteric, and cerebral arteries), which is accompanied by hypersensitivity or anaphylaxis encompassing many organs, including the skin, respiratory, and vascular system. It has a significant impact on the mortality of patients [4]. It is believed that even up to 13% of sudden cardiac deaths in adults are connected with MC degranulation in the progression of this syndrome [24,81]. Until now, it was associated mainly with the coexistence of acute coronary syndromes and conditions connected with the activation of MCs, including anaphylaxis or pseudoanaphylaxis [24].

As a result of the release of inflammatory mediators by activated MCs, the coronary artery spasms and/or the atherosclerotic plaque breaks off with clinical symptoms of acute coronary syndrome or myocardial infarction, as well as cerebrovascular symptoms [4,24]. This mechanism may explain why the concentrations of histamine, neutral proteases, arachidonic acid metabolites, platelet activating factor, and chemokines are increased in the blood or urine both during allergic episodes and in acute cardiac syndromes. There is evidence that MCs do not have to be located in the heart, but it is the result of their mediators being released before the actual cardiac episode [4,24]. A 20% platelet subgroup with IgE surface receptors with high and low affinity also takes part in this process [4].

There are three variants of the Kounis syndrome:

type I—patients with normal coronary arteries without factors predisposing them for coronary disease, in whom an acute allergic reaction causes a spasm of the coronary artery with normal cardiac enzymes and troponins, or a coronary artery vasospasm leading to acute myocardial infarction with increased cardiac enzymes and troponins (symptom of endothelium dysfunction or microvascular angina);
type II—patients with pre-existing atherosclerosis, in whom an acute allergic episode may cause the erosion or breaking off of plaque, manifested as an acute myocardial infarction [4,24,82];
type III—patients with thrombosis in the coronary stent or restenosis of the stent due to an allergic inflammation (stent elements containing nickel may act as allergens causing secondary post-thrombotic changes) [4,83].

The cause behind the Kounis syndrome may be various drugs (most frequently acetylsalicylic acid), food (e.g., raw fish infected by the *Anisakis simplex* nematode, seafood and scombroid food poisoning), latex, environmental exposure, and clinical conditions. [82,83,83]. Scombroid food poisoning consists in the poisoning of fish with Gram-negative bacteria containing the enzyme histidine decarboxylase which converts the amino acid histidine (contained in the fish) into histamine and then triggers the Kounis syndrome symptoms [83].

The confirmation of the Kounis syndrome diagnosis is not easy. The tryptase level should be monitored (every thirty minutes from the first symptoms and then every 30 min for the next 2 h), as well as the histamine (present for just 8 min after the allergic event), IgE, cardiac enzymes, and cardiac troponins in the blood serum [4,84].

The therapeutic procedure is difficult because its aim is to ensure revascularization of the cardiac muscle, as well as the treatment of the coexisting allergic reaction. There are no explicit guidelines. Corticosteroids, e.g., hydrocortisone in the dose of 1–2 mg/kg/day, result in stopping the hyperreactivity of arteries and have an anti-inflammatory effect, while antihistamine drugs H1 and H2 have an antiallergenic effect. Moreover, patients require appropriate fluid resuscitation, particularly as vasodilatory drugs should be administered simultaneously (calcium channel blockers, nitrates); the purpose of which is to alleviate the vascular spasm caused by hypersensitivity [82]. Epinephrine should be used cautiously because it can exacerbate the myocardial ischemia, extend the QTc interval, and cause a spasm of cardiac vessels and cardiac arrythmia [82,85].

## 8. Role of Mast Cells in Ehlers–Danlos Syndrome (EDS)

Ehlers–Danlos syndrome (EDS) is a hereditary group of collagen and extracellular-matrix protein disorders. This syndrome has a heterogenous etiology and is mostly characterized by joint hypermobility, skin hyperextensibility, and tissue fragility. According to the 2017 EDS classification, there are 13 different variants. The type of EDS is determined by genetic testing, but up to 50% of patients can have a de novo mutation. The genetic etiology is known for most EDS subtypes, excluding hypermobile EDS (hEDS), which is the most common variant [86]. The prevalence of hEDS is estimated to be between 1 in 10,000 and 1 in 15,000 [87].

There is an abundance of MCs in the connective tissue near nerves, lymph nodes, and blood vessels, and this in combination with their ability to secrete strong mediators may lead to connective tissue disorders (CTDs) [88]. A possible connection between hEDS and MCAS has been observed. This type constitutes 40% of all EDS cases, and still no specific, highly recurring reason behind the mutation has been identified [88,89,90].

Immunohistochemical tests revealed a higher content of chymase-positive MCs in the undamaged skin of patients with CTD symptoms (skin hyperelasticity, joint hyperpermeability, spinal and chest deformations, symptoms in the thumb and wrist, brittle blood vessels, varicose veins, and telangiectasias) [88,90]. MCAS is probably linked to the incorrect expression of the MC mediator, caused by mutations in MC regulatory elements (e.g., genes, microRNAs) [89,91]. Hypothetically, the connective tissue proteins may initially have the correct structure, but possible abnormal stimulation by substances released by MCs can lead to the destruction of tissue and to the onset of hEDS [89]. However, the data are still insufficient.

## 9. Role of Mast Cells in Exanthemas in the Course of Hypersensitivity Reaction to Drugs

### 9.1. Anaphilactoidal Reaction (Pseudoallergy)

The participation of MCs in the reaction to drugs usually manifests as non-immunologic anaphylactoid reactions (idiosyncrasies), constituting a frequent cause of MCAS. Currently, the name “pseudoallergy” is being proposed. Such reactions are often caused by the first dose of the drug, and this is the reason for their unpredictability and sometimes even fatal consequences [92]. Clinical symptoms develop in the form of urticaria, angioneurotic oedema and anaphylaxis, bronchial spasm, gastrointestinal symptoms, including erythema, headache, oedema, hypotension, and shock. Clinical symptoms of pseudoallergy and anaphylaxis are clinically indistinguishable. Interestingly, patients with MCAS have various food intolerances not connected with IgE, which cause various symptoms. On the other hand, the very presence of MCAS may increase the severity of allergic reactions, which was demonstrated in the study of patients with MCAS and an allergy to amoxicillin [93,94].

There are two mechanisms of this reaction: one connected with the activation of the complement and one connected with the activation of MCs [92]. In the case of the latter mechanism, this takes place as a result of the suppressing of cyclooxygenase 1, activation of the MC surface receptor Mrgprb2, which is the ortholog (i.e., homolog of a protein or a DNA/RNA fragment with a shared evolutionary origin) receptor coupled with G MRGPRX2 (non-IgE-mediated Mas-related G-protein-coupled receptor member X2) [28,95]. (Table 2). Other effects of the non-immunologic MC degranulation is the release of histamine, activation of the complement system, and suppression of the bradykinin degradation [92].

In the case of hypersensitivity to drugs with accompanying MCAS, the rapid desensitization (RD) method may be used if, due to the disease, the patient requires the particular drug. This is done in the case of pregnant women allergic to penicillin who have to be treated for syphilis. The fact that there is no alternative treatment as macrolides do not prevent the transmission of spirochaetes to the fetus and tetracyclines may result in fetal abnormalities constitutes the reason to use this procedure [96]. The recommendations of the Center for Disease Control and Prevention in 2015 contained in the Sexually Transmitted Diseases Treatment Guidelines indicate this explicitly [97]. Similar recommendations have been presented in the latest European Dermatology and Venereology guidelines as the first-line regimen [98]. The RD protocols have been developed and are available online [99]. The procedure must be conducted in specialized centers in accordance with the protocol developed in order to obtain tolerance to the drug which causes anaphylactoid (IgE-independent) or anaphylactic reactions (IgE-dependent).

Desensitization consists in administering successively increasing doses every 15 to 30 min, until the achievement of clinical tolerance to the full therapeutic dose which causes the stabilization of basophiles and MCs. The mechanism has not been fully understood, but it is believed to be the result of the subthreshold stimulation of the antigen by the hapten–carrier conjugate [100]. Staso et al. described the case of a patient with MCAS and a history of general systemic reactions to ceftriaxone and azithromycin who required the administration of these drugs to treat pneumonia [100].

RD may be performed not just for beta-lactam antibiotics but also in the case of sulfonamides and other antibiotics, as well as aspirin. One should remember that desensitization in the case of hypersensitivity to drugs in the course of MCAS is a complicated and controversial process because the hypersensitivity mechanism may not be connected with nonspecific MC degranulation [100].

### 9.2. Red Man Syndrome

Another drug-induced reaction in which MCs participate is the red man syndrome. Some patients have a reaction in which a large quantity of histamine is released from MCs and basophile in response to vancomycin through the interaction with receptor MRGPRX2 [101,102].

Red man syndrome was initially described as a reaction to the fast infusion of the first dose of vancomycin and explained by the response to contaminants in the preparation. However, this reaction has been also observed in the case of other antibiotics (e.g., ciprofloxacin, amphotericin B, rifampicin, and teicoplanin), opioid analgesics, muscle relaxants, and contrast media [102,103,104]. A similar reaction after infliximab infusion has also been described in as many as 5% to 23%, finding the presence of antibodies to the drug in this group; therefore, this reaction may be of a more complex nature. Besides the histaminergic path, alternative inflammatory paths take part which may include the release of histamine [104,105,106]. The prevention of this reaction consists in the slow intravenous infusion of these drugs, with possible administration of diphenhydramine or H1 receptor blockers with the H2 receptor blocker (cimetidine, ranitidine) [102].

The symptoms of red man syndrome appear around 4–10 min after starting the infusion or may begin after the infusion has ended. The symptoms manifest as pruritus, burning, and exanthema covering the face, neck, and upper body. Rarer symptoms include hypotension and angioneurotic oedema [102,103,105]. There may be a quick onset of vertigo and agitation, as well as headache, shivering, fever, and paresthesia around the mouth, and in severe cases, even chest pain and dyspnea [102].

Myers et al. identified factors predisposing patients to the red man syndrome for vancomycin: Caucasian race, age ≥ 2, medical history of previous syndrome, vancomycin dose ≥ 10 mg/kg, its concentration ≥ 5 mg/ml, and, which is interesting, earlier use of antihistamine drugs. However, genetic variants of histamine metabolism or receptors were of no greater significance [105].

## 10. Role of Mast Cells in Allergic Contact Dermatitis

Allergic contact dermatitis (ACD) is an inflammatory reaction of the skin, occurring as a result of the abnormal response of the immune system to external substances. ACD arises as a consequence of type IV hypersensitivity resulting from the activation of T lymphocytes specific to the allergen. The allergen is a hapten, i.e., an antigen with a small particle mass which, after being attached to a larger carrier (protein or polysaccharide), becomes immunogenic. The hapten–carrier complex, thanks to dendric cells, is carried to regional lymph nodes, and there the presentation of the antigen to T cells takes place. Another exposure of the skin to the allergen activates T cells specific to the hapten, reactivating proinflammatory cytokines and intumescence of immune system cells, including MCs [107]. The porousness of the epidermal barrier creates favorable conditions for the development of ACD. This happens in the case of AD, where the effect of proteins building the cytoskeleton of keratinocytes occurs, e.g., filaggrins, as well as in the case of chronic dermatitis, e.g., in the course of chronic leg ulcers, which directly promotes potential allergens entering the epidermis [107]. In ACD and AD, activation of the endothelium cells stimulates MCs to release vasoactive compounds and proinflammatory cytokines [108]. Studies of the delayed hypersensitivity of mice with MC deficiency indicate a weaker allergenic and inflammatory response, which may confirm the key role of MCs in the pathogenesis of allergic diseases [109]. Contact allergens, on one hand, release immediate degranulation of MCs, causing the release of granularity, which results in the occurrence of ACD symptoms—the pruritic, erythematous papular-vesicular rash. On the other hand, contact allergens stimulate MCs to produce and release late proinflammatory mediators (e.g., IL-4, Il-5, Il-6, Il-13, and TNF-alfa), cytokines from the Il-1 family, and chemokines (e.g., CCL2, CCL3, and CCL4), which sustains and stimulates the inflammation [110].

## 11. Role of Mast Cells in Rosacea

MCs take part in the pathogenesis of rosacea, but their role has not been fully explained yet (Table 3). The intensification of clinical changes is positively correlated with the number of MCs in the skin, as well as with the duration of the condition [111,112]. Moreover, MCs were located mainly around vessels, and the expression of the mRNA of chymase and metalloprotease 9 (MMP9), basic markers of the presence and activation of MCs, was significantly increased in the skin with rosacea in comparison with healthy skin [112,113,114].

Both active antimicrobial peptide LL-37 and immune-cell-expressed proteinase-activated receptor-2 (PAR 2) affect the activity of MCs, leading to degranulation and the release of proinflammatory cytokines (IL-6) and metalloproteinase MMP-9 [113,114,115]. Moreover, MCs produce proangiogenic molecules (VEGF, fibroblast growth factor—FGF, histamine and tryptase), which leads to angiogenesis and constitutes the reason behind numerous telangiectasias and erythema, as well as the increased permeability of vessels in the skin [112,114].

Most recent research emphasizes the role of MCs, particularly in the phymatous clinical variants of the disease (phyma), through the production of metalloproteinases and FGF. A correlation between the number of MCs and the duration of the disease has been shown, which points to their participation in sustaining the inflammatory process [114].

## 12. Cutaneous Mastocytosis

### 12.1. Clinical Presentation of Mastocytosis

Mastocytosis is a clonal hematopoietic disease, characterized by the proliferation and accumulation of morphologically and immunophenotypically abnormal MCs in one or more organ systems [116,117,118]. The World Health Organization (WHO) classification of mastocytosis (2016) distinguishes three major forms of the disease: cutaneous mastocytosis (CM), which is a skin-limited disease, SM, and MC sarcoma (MCS) [116,119]. Activating mutations of *KIT* (D816V *KIT* mutation in the majority of patients with SM), which leads to the excessive proliferation of MCs and the subsequent accumulation of these cells in tissues, plays a crucial role in the pathogenesis of mastocytosis [117,118,120,121]. MCs may infiltrate only the skin (CM) or extra-cutaneous organs, including the bone marrow, spleen, liver, lymph nodes, and gastrointestinal tract (SM with or without skin involvement) [116,117,118,119,121,122,123]. The clinical presentation of mastocytosis depends on both the infiltration of various organs by MCs and the mediators released by these cells as a result of IgE-mediated or non-IgE-mediated activation of MCs, making the disease heterogeneous.

CM may present as maculopapular CM (MPCM), diffuse CM (DCM), and mastocytoma of the skin [122]. Most patients with CM suffer from the MPCM subtype, which is characterized by round brown or red lesions. In the majority of children, skin lesions are larger than in adults and present as macules, papules, or nodules, with asymmetric distribution, typical for a polymorphic variant of MPCM. Unlike in children, the monomorphic variant of MPCM with typically small, mostly macular lesions dominates in adults [117]. DCM due to MC infiltration over the entire skin is a rare form of CM, often associated with severe MC mediator-related symptoms related to the significant burden of MCs. DCM manifests clinically as erythroderma and generalized pachydermia (thickened skin) with occasional papules on the surface and typically intense and prolonged dermographism. A characteristic feature of DCM in infancy is the presence of severe blisters filled with serous or hemorrhagic fluid, which may appear in response to mechanical irritation of the skin [117]. Cutaneous mastocytoma (single or up to three lesions) is the mildest form of CM, which usually appears in infants. It presents as brown, red, or yellow macules or nodules, sharply demarcated from the surrounding area and shows the Darier’s sign after rubbing [121].

### 12.2. Mast Cell Mediator-Related Symptoms in Patients with Mastocytosis

The most common mediator-related symptoms experienced by patients with CM include Darier’s sign, pruritus, flushing, blistering, headaches, diarrhea, vomiting, abdominal pain, hypotension, dizziness, bronchospasm, and anaphylactic shock [117,118,119,120,121,122,123,124,125,126,127,128,129,130,131,132,133,134,135,136,137,138]. In both CM and SM patients, MCs produce, among others, histamine, heparin, tryptase, carboxypeptidase, chymase, lysosomal enzymes, numerous cytokines, chemokines, growth factors, prostaglandin D2 (PGD2), leukotriene C4 (LTC4), platelet-activating factor (PAF), and sphingosine-1-phosphate [39,54,139,140,141,142]. Moreover, it was found that *KIT* D816V-transformed MCs display oncostatin M (OSM), IL-8, IL-6, chemokine ligand 2 (CCL2), and CCL23 [54,139,142,143]. MC-derived OSM stimulates the growth of microvascular endothelial cells, osteoblasts, and fibroblasts [139]. Furthermore, it was shown that in children with mastocytosis, MCs may migrate to the skin and BM as a result of the upregulation of CCL2/chemokine receptor 2 (CCR2) and vascular cell adhesion molecule (VCAM-1), and they may induce the expression/activation of transglutaminase 2 (TG2) [144]. Importantly, TG2 is a cross-linking enzyme that promotes the expression of inflammatory cytokines, histamine, and LTC4 [144]. An assessment of the precise role of all MC mediators in the pathogenesis of mastocytosis requires further studies.

Darier’s sign is a pathognomonic feature of CM defined by the whealing and reddening of lesions upon mechanical irritation of the skin lesion. It is elicited by stroking the lesion (approximately five times) using moderate pressure with a tongue spatula [122]. A positive Darier’s sign is confirmed if a wheal-and-flare reaction of the lesion develops within a few minutes [122]. This local reaction is mainly related to histamine released from MCs [39,54].

Pruritus, defined as an unpleasant feeling that evokes a desire to scratch, which negatively affects psychological and physical aspects of life, is one of the most common cutaneous symptoms in mastocytosis [130]. The pathogenesis of pruritus in mastocytosis is still unclear; however, the participation of MCs in neurogenic inflammation is emphasized [129]. Neurogenic inflammation is connected with anatomical association due to MC distribution in the dermis, especially in perivascular areas. In the process of intercellular communication, mediators released by MCs activate receptors on nerve endings, thereby modulating the functioning of sensory nerves. The stimulation of the sensory nerves leads to the expression of neuropeptides which increases neurogenic inflammation and associated pruritus [129]. The mutual interactions between MCs and peripheral nerves are enhanced by the expression of the same receptors and additionally by the ability of both of these cells to respond to stimulation by the same mediators [129]. Pruritogenic neurotransmitters, such as chymase, tryptase, serotonin, histamine, and interleukins (including interleukin-31), are released from dermal MCs in response to different stimuli [129].

Blistering is a typical symptom of a pediatric CM which is observed in early childhood (in approximately 25–35% of children) [122]. Bullous lesions occur mainly in children with extensive skin involvement (usually in DCM) and may present as generalized, recurrent bullous changes or small-vesicular changes on the basis of erythroderma [135]. Blistering can be provoked by physical stimulation such as a mechanical irritation and non-specific factors like infection and immunization; it may also develop spontaneously [131]. The serine proteases released from MCs by the proteolytic activity in the lamina lucida separate the epidermis from the basement membrane, which leads to junctional blistering [135].

Flushing symptom is characterized by a sudden, confluent erythema of the face, neck, and possible extension to the entire surface of the skin. It is caused by a vascular response to MC mediators [122]. The dilatation of the dermal capillaries is secondary to vasoactive mediators, such as histamine, prostaglandins (e.g., PGD2), serotonin, and SP [122]. Massive flushing may predispose to the development of rapid, potentially life-threatening hypotension due to generalized cutaneous vasodilation caused by a rapid release of mediators from MCs [122,138].

## 13. Discussion

In the pathophysiology of skin diseases, three groups of MCs can be distinguished due to the prevailing mechanisms:

- “cancerous MCs” associated with clonal proliferation (mastocytosis group) [122];
- “overactive MCs” activating allergic processes (atopic dermatitis, urticaria, allergic contact dermatitis, hypersensitivity reaction to drugs, MCAS) [4,77,92,145];
- “neurogenic MCs” associated with neuroinflammation in diseases exacerbated by stress (psoriasis, atopic dermatitis, urticaria, rosacea) [74,112,146].

So far, the best-known pathophysiological mechanisms of MCs in skin diseases are those related to their clonal neoplastic growth (mastocytosis group), as well as allergic diseases proceeding in the IgE-dependent mechanism (allergies to foods, pollen, drugs, etc.) [19,38,131,140]. In recent years, more and more papers have been published indicating the key role of MCs in inflammatory skin diseases, including those of autoimmune origin [13,147]. Due to the lack of sufficient data, the role of MCs in inflammatory skin diseases is not clear and requires further research. Difficulties in studying the role of MCs in inflammatory skin diseases may be due to many reasons. First of all, MCs are called rapid response cells, and the mechanism of their activation may not always be captured, especially in biopsies from long-term skin lesions. Moreover, MCs participate in an extremely complex network of connections with other cells of the immune system but also with other cells of the skin and epidermis; the most reliable studies would be performed in vivo in humans, which is unlikely. In addition, there is also no human “mast-cell-free” model, which could indicate which processes in the human body would not function without MCs.

## 14. Conclusions

MCs play an important immunomodulatory role by releasing numerous proinflammatory and anti-inflammatory mediators, thanks to which the immune system has the ability to respond quickly to changing environmental conditions. The confirmation of this fact is that a great number of MCs are found in the skin and in the digestive and respiratory systems, i.e., systems and organs which are in direct contact with the external environment. Besides their physiological role, MCs participate in numerous pathological processes including allergies, tumors, or inflammatory skin conditions.

Taking into account the fact that MCs have many receptors for various signaling pathways on their surface and are capable of secreting many cytokines themselves, it can be said that their proper function is the creation of a microenvironment for other cells of the immune system. However, immunological processes are multifactorial. Therefore, determining the function of individual cells, cytokines, and chemokines may carry the risk of incorrect conclusions.

Symptoms arising as a result of the activation of MCs constitute components of MCAS. Due to the diversity of symptoms and the possibility of many organs being affected, diagnosing MCAS may be difficult for many specialists. Therefore, in order to improve the diagnostics and treatment of this complex pathology, multidisciplinary cooperation and further studies on the role of MCs are required. Moreover, due to the important role of MCs in many skin diseases, suppressing their activity is believed to be a promising treatment strategy in inflammatory skin diseases.

## Figures and Tables

**Table 1:** Factors predisposing to the development of MCASs.

Genetic factors	Genes encode *IL-4* or *IL-6*, the production of IgE, and/or the regulation of the expression of the IgE receptor on MCs [30,31,32,33]; genes encode IL-13 [30,34]; the *KIT* mutation D816V [35,36,37]; genes encode angiotensinogen, angiotensin-converting enzymes, and chymase [38,39,40,41]; extra copies of the *TPSAB1* gene encoding for alpha tryptase [42,43]
Environmental factors	Animal venoms (e.g., Hymenoptera insect, snake, black widow spider, scorpion); hypersensitivity to food (e.g., seafood, tuna fish, salt fish, uncooked anchovies, scombroid, food poisoning); alcohol; medications (e.g., antibiotics, non-steroidal anti-inflammatory drugs (NSAIDs), morphine, neuromuscular blocking agents, radiocontrast media, general anesthesia); physical stimuli (e.g., biopsy, endoscopy, vibration, exercise, pressure, friction, heat); infections (e.g., Ebstein–Barr virus (EBV), varicella zoster virus (VZV), herpes simplex virus (HSV), cytomegalovirus (CMV), human immunodeficiency virus (HIV), influenza, dengue, Ebola, Helicobacter pylori, helminthic parasites); stress [44,45,46,47,48,49,50].
Comorbidities	HαT, SM, cutaneous mastocytosis, IgE-dependent allergy (especially Hymenoptera venom allergy) or other hypersensitivity, atopic disease, vibration urticaria, chronic inflammatory disorders, auto-immune disorders, rheumatic diseases, neurologic diseases, infectious disease [39,40,51,52,53,54,55]

**Table 2:** Mechanisms of MC activation by medication [92].

Type of Medication	Mechanism of MC Activation
Acetylsalicylic acid, NSAID	Inhibition of cyclooxygenase-1
Neuromuscular blocking agents, vespid mastoparan, opioids, icatibant, all fluoroquinolones	Activation of MRGPRX2
Vancomycin, iodinated contrast media	Unknown
Liposomal drugs: AmBisome and Doxil, and the micelle-solubilized drug Taxol	Activating the complement system

**Table 3:** The role of MCs in rosacea [112].

Element of Pathogenesis	Mechanism	Effect
innate immune responses	a production of LL-37 cathelicidin and LL-37 stimulate MCs to release IL-6 and MMP9	promote protease production and amplify inflammation
neurogenic inflammation	stimulation of MCs by neuropeptides (serotonin receptor 3A, pituitary adenylate cyclase-activating polypeptide, SP, vasoactive intestinal peptide) can cause MC degranulation and the release of histamine, tryptase, and other mediators (TNF-α, CXCL9, CXCL10, CXCL8)	promoting inflammation resulting in itching, flushing, erythema, and burning sensations
vasodilation and angiogenesis	a production of proangiogenic molecules (VEGF, fibroblast growth factor—FGF, histamine, and tryptase)	stimulation of the migration and proliferation of endothelial cells, leads to facilitating vascularization and angiogenesis;
		histamine and serotonin can bind to the vascular receptors, causing vasodilation and increased vascular permeability; vasodilation leads to erythema, and increased vascular permeability causes flushing
fibrosing	histamine and tryptase enhance fibrosis through their chemotactic function on fibroblasts and MMPs; the proliferation of fibroblasts through VEGF and basic FGF	manifestation of phyma

## Data Availability

Not applicable.
